# An Efficient Procedure for Marker-Free Mutagenesis of *S. coelicolor* by Site-Specific Recombination for Secondary Metabolite Overproduction

**DOI:** 10.1371/journal.pone.0055906

**Published:** 2013-02-07

**Authors:** Bo Zhang, Lin Zhang, Ruixue Dai, Meiying Yu, Guoping Zhao, Xiaoming Ding

**Affiliations:** 1 Department of Microbiology and Microbial Engineering, School of Life Sciences, Fudan University, Shanghai, China; 2 Key Laboratory of Synthetic biology, Institute of Plant Physiology and Ecology, Shanghai Institutes for Biological Sciences, Chinese Academy of Sciences, Shanghai, China; 3 Shanghai-MOST Key Laboratory of Health and Disease Genomics, Chinese National Human Genome Center at Shanghai, Shanghai, China; 4 Department of Microbiology and Li Ka Shing Institute of Health Sciences, The Chinese University of Hong Kong, Prince of Wales Hospital, Shatin, New Territories, Hong Kong SAR, China; Institut Pasteur Paris, France

## Abstract

*Streptomyces* bacteria are known for producing important natural compounds by secondary metabolism, especially antibiotics with novel biological activities. Functional studies of antibiotic-biosynthesizing gene clusters are generally through homologous genomic recombination by gene-targeting vectors. Here, we present a rapid and efficient method for construction of gene-targeting vectors. This approach is based on *Streptomyces* phage φBT1 integrase-mediated multisite *in vitro* site-specific recombination. Four ‘entry clones’ were assembled into a circular plasmid to generate the destination gene-targeting vector by a one-step reaction. The four ‘entry clones’ contained two clones of the upstream and downstream flanks of the target gene, a selectable marker and an *E. coli-Streptomyces* shuttle vector. After targeted modification of the genome, the selectable markers were removed by φC31 integrase-mediated *in vivo* site-specific recombination between pre-placed *attB* and *attP* sites. Using this method, part of the calcium-dependent antibiotic (CDA) and actinorhodin (Act) biosynthetic gene clusters were deleted, and the *rrdA* encoding RrdA, a negative regulator of Red production, was also deleted. The final prodiginine production of the engineered strain was over five times that of the wild-type strain. This straightforward φBT1 and φC31 integrase-based strategy provides an alternative approach for rapid gene-targeting vector construction and marker removal in streptomycetes.

## Introduction

Members of the Gram-positive, spore-producing genus *Streptomyces* play critical roles in soil ecology and are useful for synthesizing medically and industrially important secondary metabolites. Genomic sequencing of the model organism *Streptomyces coelicolor* A3(2) was completed in 2002 [1], which dramatically facilitated gene function studies of these bacteria, especially those involved in antibiotic production through targeted modification [2]. Additionally, with the increased data of whole genomic actinomycetes sequences, robust and rapid methods are required to study the functions of a large number of important genes. The gene-targeting approach through homologous recombination was the first step in construction of gene-targeting vectors. The traditional method of constructing vectors is by step-by-step enzymatic digestion and ligation following PCR amplification and assembly of independent DNA segments into a plasmid. However, this method is complicated and time consuming, and often limited by lack of proper restriction enzymatic sites.

Many methodologies have been developed for gene targeting vector construction [3], [4], [5], [6], especially those of recombination-based strategies [4], [5], [7], [8], [9], [10], [11], [12], [13], [14]. Among these, a powerful method, termed REDIRECT© technology (PCR-targeting system) [4], [7], [15] has been widely used in *Streptomyces*. This procedure is based on phage λ-Red proteins to promote recombination, which employs Redα (*exo*), Redβ (*bet*) and Redγ (*gam*) to mediate recombination when only tens of nucleotides are homologous to the target region [4]. In *E. coli* with helper plasmids, PCR-amplified selectable markers using primers with only 39 nt homology extensions are used to amplify chromosomal sequences within a genomic cosmid library [15] to generate the desired gene-targeting vectors. Another universal cloning method, referred to as Gateway® technology [8], is based on phage λ site-specific integrase between *attB* and *attP* sites and excision between *attL* and *attR* sites. Using expanded properties of recombination sites with unique specificities, many segments could be cloned into a vector backbone [16], thereby expediting several simple one-week methods to construct gene-targeting vectors [5], [12], [14]. These two phage λ-recombination-based technologies are simple and efficient, and have been combined together for constructing gene targeting vectors to generate knock-out mice [17]. Furthermore, the μ transposon was developed to allow random insertion of selectable markers and other desired sequences into destination plasmids for rapid generation of gene-targeting vectors [3], [18], [19], which complemented existing recombination-based approaches for generation of gene-targeting constructs [3].

We previously established a highly efficient *in vitro* site-specific recombination system based on *Streptomyces* phage φBT1 integrase and identified the minimal sizes of *attB* and *attP* sites (36-bp and 48-bp, respectively), which was smaller than that of λ site-specific recombination (25-bp and 200-bp, respectively) [20]. We selected 16 pairs of non-compatible recombination sites, of which the central dinucleotides were not identical, and inhibited DNA strand exchange and religation, thus no recombination could occur between site pairs containing different core sequence mutations [21]. Here, we report a simple and highly efficient system for marker-free gene targeting in *S. coelicolor* by combining φBT1 integrase-mediated multisite recombination *in vitro*, homologous recombination and φC31 integrase-mediated site-specific recombination *in vivo* [22]. This simple strategy should be readily suitable and advantageous for poorly genetically established *Streptomyces* systems without an ordered cosmid library, coupled with a desire to knockout longer DNA segments, and could be easily adopted to other organisms to construct gene-targeting vectors.

Using this method, we constructed an *S. coelicolor* strain for overproduction of prodiginine (Red), which is one of the four main antibiotics produced by *S. coelicolor* A3(2) with anti-fungal, anti-bacterial, anti-protozoan, anti-malarial, immunosuppressive and anti-cancer activities [23], [24]. The biosynthesis of calcium-dependent antibiotic (CDA) and actinorhodin (Act), which might influence Red production by competition of common precursors, were disrupted by homologous recombination after parts of the key genes of these two biosynthetic clusters were deleted. For CDA, nonribosomal peptide synthetase (NRPS) coding genes *cdaPS1*, *cdaPS2* and part of *cdaPS3*, were replaced by the apramycin resistance gene *aac(3)IV*; and for Act, structural genes (*actIII* to *actVB*) were deleted. In addition, one of the TetR family protein genes, *rrdA*, which negatively regulates Red production by controlling the abundance of RedD mRNA [25], was also deleted using our method. The final Red production of the engineered strain was over five times that of the wild-type strain.

## Materials and Methods

### Bacterial strains, plasmids, and growth conditions

The bacterial strains and plasmids used in this work are listed in Table 1. *E. coli* DH5α [26] was used for plasmid propagation. Mannitol soy flour [27] agar was used to generate spores and select for *S. coelicolor* exconjugants. R2YE agar was used for phenotype screening. R4 liquid medium (100 mg casamino acids, 1 g yeast extract, 3 g proline, 10 g MgCl_2_·6H_2_O, 10 g glucose, 4 g CaCl_2_·2H_2_O, 5.6 g TES, and 0.2 ml trace element in 1.0 L H_2_O) was used for antibiotic production. The conjugal transfer from *E. coli* ET12567/pUZ8002 into *S. coelicolor* was performed as described previously [27]. *Bacillus mycoides* Flugge ATCC 6462 was used as indicator strain for CDA production assay [27]. Antibiotics were added at the following final concentrations: ampicillin, 50 µg ml^−1^; apramycin, 30 µg ml^−1^; chloramphenicol, 34 µg ml^−1^; kanamycin, 30 µg ml^−1^; and thiostrepton, 20 µg ml^−1^.

**Table 1:** Strains and plasmids used in this study.

Strains or plasmids	Genotype or description	Ref. or source
***S. coelicolor***		
M145	SCP1- SCP2-	[27]
ZB1	M145 with CDA gene cluster disrupted, containing *aac(3)IV* gene copy in the chromosome	This work
ZB2	ZB1 with the resistance gene removed	This work
ZB3	ZB2 with Act gene cluster disrupted, containing a *aphII* gene copy in the chromosome	This work
ZB4	ZB3 with the resistance gene removed	This work
ZB7	ZB4 with *rrdA* gene disrupted, containing a *aphII* gene copy in the chromosome	This work
ZB8	ZB7 with the resistance gene removed	This work
ZB8/pFDZ16-*rrdA*	ZB8 carrying integrative plasmid pFDZ16-*rrdA*	This work
ZB8/pFDZ16	ZB8 carrying integrative plasmid pFDZ16	This work
***E. coli***		
DH5α	F- *recA lacZ* ΔM15	[26]
ET12567	*dam dcm hsdS*	[27]
**Plasmids**		
pMD19-T	2.7-kb cloning vector; Amp^r^	Takara
pBC-AM	Donor of *aac(3)IV*; Apra^r^ Cm^r^	[25]
pHZ1358	*E. coli*-*Streptomyces* shuttle vector; Amp^r^ Thio^r^	[25]
pXD34-int	*E. coli*-*Streptomyces* shuttle vector; derivative obtained from pIJ6021, containing *tipA* promoter and the φC31 integase gene; Apra^r^ Thio^r^ Kan^r^	This work
pFDZ100	*E. coli*-*Streptomyces* shuttle vector; derivative obtained from pHZ1358, containing *attP*_0_ site and *attB*_15_ site; Thio^r^ Amp^r^ Cm^r^	This work
pTA0006	Derivative obtained from pMD19-T, containing *attB*_0_ site and *attP*_6_ site; Apra^r^	[32]
pTA0613	Derivative obtained from pMD19-T, containing *attB*_6_ site and *attP*_13_ site; Apra^r^	[32]
pTA1315	Derivative obtained from pMD19-T, containing *attB*_13_ site and *attP*_15_ site; Apra^r^	This work
pFDZ101	Derivative obtained from pTA0006, containing *attB*_0_-φC31 site	This work
pFDZ102	Derivative obtained from pTA0613, replacing the *aac(3)IV* resisitance gene with the *aphII* resistance gene	This work
pFDZ103	Derivative obtained from pTA1315, containing *attP*_0_-φC31 site	This work
pFDZ101-CDA-5′arm	Derivative obtained from pFDZ101, containing upstream homologous arm of CDA biosynthetic gene cluster gene-targeting	This work
pFDZ103-CDA-3′arm	Derivative obtained from pFDZ103, containing downstream homologous arm of CDA biosynthetic gene cluster gene-targeting	This work
pFDZ101-Act-5′arm	Derivative obtained from pFDZ101, containing upstream homologous arm of Act biosynthetic gene cluster gene-targeting	This work
pFDZ103-Act-3′arm	Derivative obtained from pFDZ103, containing downstream homologous arm of Act biosynthetic gene cluster gene-targeting	This work
pFDZ101-*rrdA*-5′arm	Derivative obtained from pFDZ101, containing upstream homologous arm of *rrdA* gene	This work
pFDZ103-*rrdA*-3′arm	Derivative obtained from pFDZ103, containing downstream homologous arm of *rrdA* gene	This work
pFDZ100-CDA-tandem	Derivative obtained from pFDZ100, containing two homologous arms of CDA biosynthetic gene cluster gene-targeting; Apra^r^	This work
pFDZ100-Act-tandem	Derivative obtained from pFDZ100, containing two homologous arms of Act biosynthetic gene cluster gene-targeting; Kan^r^	This work
pFDZ100-*rrdA*-tandem	Derivative obtained from pFDZ100, containing two homologous arms of *rrdA* gene; Kan^r^	This work
pZB101	*E. coli*-*Streptomyces* shuttle vector; derivative obtained from pHZ1358, containing *tipA* promoter and the φC31 integase gene; Kan^r^ Thio^r^ Amp^r^	This work
pZB102	*E. coli*-*Streptomyces* shuttle vector; derivative obtained from pZB101, replacing the *aphII* resisitance gene with the *aac(3)IV* resistance gene	This work
pSET152	Integrative vector for actinomycetes; containing *oriT*, *int*, and *attP*-φC31 site, Apra^r^	[27]
pRT802	*E.coli-Streptomyces* shuttle plasmid, encoding φBT1-int and *attP*, resistant to kanamycin	[28]
pFDZ16	*E. coli-Streptomyces* integrative shuttle vector containing *tipA* promoter, Kan^r^, Thio^r^, Amp^r^.	[25]
pFDZ16-*rrdA*	Derivative obtained from pFDZ16, containing the *rrdA* gene located downstream of the *tipA* promoter, Kan^r^, Thio^r^, Amp^r^.	[25]

### Construction of the plasmids

**Figure 1:**
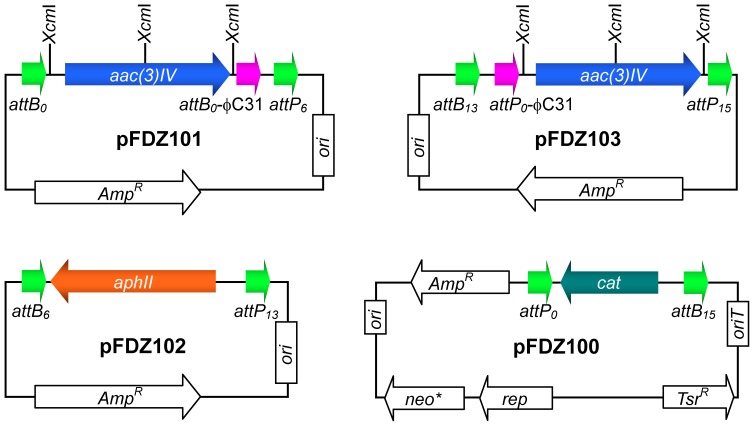
Map of four ‘entry vectors’ used in this study. Plasmids pFDZ101 and pFDZ103 were used to harbor the 5′-arm and 3′-arm respectively, and pFDZ102 contained the selectable marker (*aphII* gene). pFDZ100 was the backbone of the gene-targeting vector. Each vector contained a pair of non-compatible recombination φBT1 integration sites (arrows in green). The pink arrows represent the recombination φC31 integration sites, where recombination occurred to remove the marker gene.

All plasmids used in this study are described in Table 1, and primers are listed in Table S1. To construct the ‘entry vectors’, three 1.0 kb cassettes, *attB_0_*-*aac(3)IV*-*attP_6_*, *attB_6_*-*aac(3)IV*-*attP_13_*, and *attB_13_*-*aac(3)IV*-*attP_15_*, were PCR-amplified using the PrimeStar® PCR Kit (TaKaRa, Kyoto, Japan) and ligated into the pMD19-T vector (TaKaRa) to generate plasmids pTA0006, pTA0613 and pTA1315 (see Table 1). Primers ZB153 and ZB154 (containing *attB_0_*-φC31), ZB155 (containing *attP_0_*-φC31) and ZB156 were used to amplify the *aac(3)IV* gene fragment, which was inserted into *Xcm*I-linearized pTA0006 and pTA1315 to generate pFDZ101 and pFDZ103. These two plasmids were used to carry homologous arms after *Xcm*I digestion (see Fig. 1). Plasmid pSET152 was used as a template for the *aac(3)IV* gene.

Primer pairs ZB129/ZB130 and ZB131/132 were used to amplify two 2.1 kb homologous arm fragments of the CDA biosynthetic gene cluster, and the PCR products were inserted into pFDZ101 and pFDZ103 to generate the pFDZ101-CDA 5′arm and pFDZ103-CDA 3′arm, respectively. Primer pairs ZB125/ZB126 and ZB127/128 were used to amplify the 2 kb upstream homologous arm and 2.2 kb downstream homologous arm fragments of the Act biosynthetic gene cluster, and the PCR products were inserted into pFDZ101 and pFDZ103 to generate the pFDZ101-Act 5′arm and pFDZ103-Act 3′arm, respectively. Using the same method, we obtained the pFDZ101-*rrdA* 5′arm and the pFDZ103-*rrdA* 3′arm, containing a 1.6 kb upstream homologous arm fragment and a 1.7 kb downstream homologous arm fragment of the *rrdA* gene, respectively, which were amplified using primer pairs ZB180/ZB181 and ZB182/ZB183.

Primers Oxj128 and Oxj129 were used to amplify the *aphII* gene fragment from pRT802 [28], which was inserted into the *Xcm*I-linearized pTA0613 to generate pFDZ102. Fragment amplifications containing the *cat* gene from pBC-AM [25] flanked by *attP_0_* and *attB_15_* were generated using the primer pair PTP00/PTB15 and the product was digested with *Xba*I/*Bam*HI and inserted into the *E. coli*-*Streptomyces* shuttle vector pHZ1358 [27], which contained the plasmid origin of transfer (*oriT*) to generate pFDZ100. pFDZ100 is very unstable in streptomycetes and is easily lost when not maintained with antibiotic selection.

**Figure 2:**
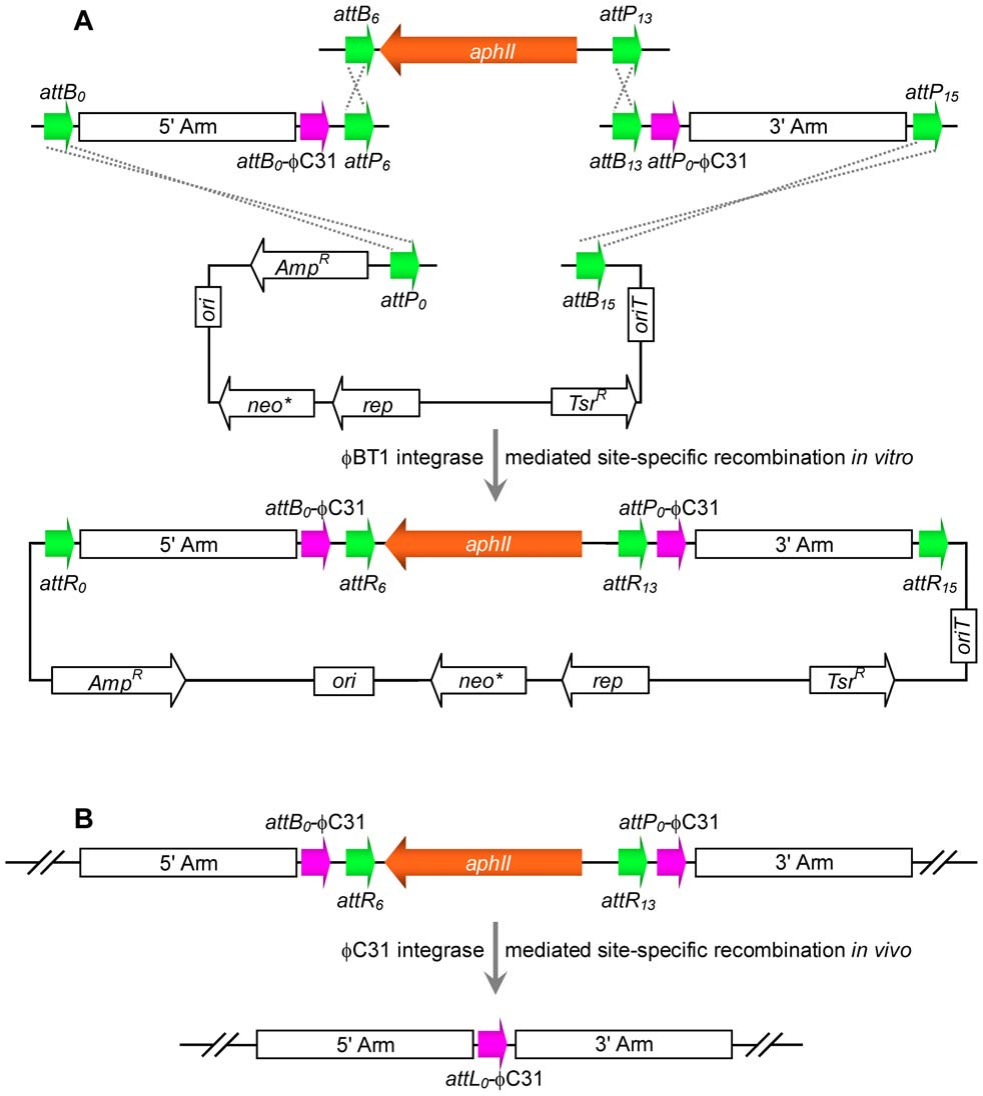
Strategy of tandem assembly *in vitro* and the marker removing *in vivo* based on site-specific recombinations. (A) One step assembly of the gene-targeting vector by φBT1 integrase-mediated site-specific recombination *in vitro*. Four recombination reactions occurred simultaneously between *attB_0_/attP_0_, attB_6/_attP_6_, attB_13_/attP_13_,* and *attB_15_/attP_15_*. (B) Deletion of marker by φC31 integrase-mediated site-specific recombination *in vivo*. The reaction occurred between *attB_0_*-φC31 and *attP_0_*-φC31 to remove the marker and leave *attL_0_*-φC31.

Construction of plasmid pXD34-int is described in the Supplemental Materials section. Plasmid pXD34-int was cut with *Eco*RV/*Nhe*I to generate a 7.1-kb fragment, which contained the *tipA* promoter and φC31 integrase gene, and then inserted into the corresponding sites in vector pHZ1358, yielding plasmid pZB101. pZB101 was further digested with *Stu*I/*Ecl*136II and then linked with a 1.5 kb *Sma*I-linearized fragment containing the *aac(3)IV* gene from plasmid pBC-AM [25] (Table 1) to generate pZB102. pZB102 was subsequently conjugated from the donor *E. coli* ET12567/pUZ8002 into the null mutants to remove the resistance gene by φC31 integrase-mediated *in vivo* site-specific recombination (see Fig. 2B). The exconjugants were selected by growth on MS media supplemented with thiostrepton (20 µg ml^−1^) and apramycin (30 µg ml^−1^). The thiostrepton was used to induce the expression of φC31 integrase. As plasmids pZB101 and pZB102 were derived from pHZ1358, which contained the *sti* DNA region (strong incompatibility locus), they could be easily lost in non-resistance stress condition [27].

### Standard in vitro recombination assays and verification of the positive assembly products

Expression and purification of φBT1 integrase was performed as described previously [20]. The recombination reaction was performed in a reaction mixture (10 µl) containing 10 mM Tris-HCl, pH 8.0, 100 mM KCl, 50 mM NaCl, 5% glycerol, integrase (4 U in 0.5 µl, ∼75 nM) and ∼30 ng DNA reaction substrates [21]. The reaction was incubated at 30°C overnight and treated with proteinase K at 55°C for 30 min and then heated to 80°C for 20 min for inactivation and transformed into *E. coli* strain DH5α. After selection with antibiotics, plasmids of positive clones were isolated and verified by enzyme digestion.

### CDA production assay

The CDA bioassay was carried out as previously described by Kieser et al. [27]. Spores were spotted onto an Oxoid nutrient agar plate and incubated for 2 days, then overlaid with soft nutrient agar containing *Bacillus mycoides* and calcium nitrate at a concentration to yield 12 mM throughout the plate. CDA produced by *S. coelicolor* killed the *B. mycoides* cells, resulting in a clearance zone on the plate.

### Quantification of Red production

Red production was assayed according to previous descriptions [27]. A culture grown in 40 ml R4 liquid medium was filtered to separate the supernatant and pellet. The mycelium pellet was collected and dried under a vacuum, then extracted with methanol overnight at room temperature. The methanol was acidified with HCl (to a final concentration of 0.5 M). Optical density was measured at 530 nm. Measurements were always performed for triplicate samples.

## Results and Discussion

### Rapid construction of the gene-targeting vectors by site-specific recombination in vitro

*Streptomyces* phage φBT1 integrase is capable of catalyzing accurate and efficient site-specific *in vitro* recombination between *attB* and *attP* sites [20]. In our previous work, 16 pairs of non-compatible sites containing different core sequences were identified, which confirmed that no recombination could occur between site pairs containing different core sequence mutations [20]. Thus, these sites could be used for multisite cloning [21], [29]. We established a multiple DNA fragment assembly method, termed site-specific recombination-based tandem assembly (SSRTA). SSRTA details and principles were described in our previous report [4], [7], [15]. As shown in Fig. 1, plasmids pFDZ101 and pFDZ103 were digested with *Xcm*I to produce 3′-T overhangs on both ends. Homologous arms could be inserted by TA cloning to generate the pFDZ101 5′-arm and pFDZ103 3′-arm. Plasmid pFDZ102 containing the *aphII* gene was used for clone selection in *E.coli* and *Streptomyces*. Plasmid pFDZ100 was used for propagation of the assembled gene-targeting vector in *E.coli* and transformed into *Streptomyces* by conjugation. Fig. 2A demonstrated the one-step tandem assembly of the four DNA fragments into a circular plasmid by φBT1 integrase-mediated *in vitro* site-specific recombination. Each fragment was flanked by a pair of non-compatible recombination sites, and after incubation with φBT1 integrase in proper buffer conditions (see Materials and Methods), the four fragments were assembled together by four simultaneous recombination events.

Using this method, we successfully constructed three gene-targeting vectors. Vectors pFDZ100-CDA-tandem and pFDZ100-Act-tandem were used to disrupt the biosynthesis of CDA and Act, respectively, and pFDZ100-*rrdA*-tandem was used for *rrdA* gene deletion. Construction details of the three vectors are in the Supplemental Information. This tandem assembly method for gene-targeting vector construction was efficient and convenient, and the resistance genes (*aac(3)IV* and *aphII* in this study) could be easily replaced by any others in pTA0613 and pFDZ102. Plasmids pFDZ101∼pFDZ103 could be *Xcm*I-digested to produce 3′-T overhangs on both ends and the homologous PCR-amplified arm fragments can be indirectly inserted by TA cloning to generate the pFDZ101 5′arm and the pFDZ103 3′arm, which avoided the limitation of available restriction recognition sites and restriction recognition sites contained in the homologous arms. Thus the construction procedure was condensed to approximately one week by our method.

### Markerless disruption of CDA, Act biosynthetic gene clusters and Deletion of the rrdA gene

Cross-regulation of endogenous gene clusters in *S. coelicolor* M145 might involve competition for common precursors [25]. Therefore, we deleted parts of two main antibiotic biosynthesis gene clusters, calcium-dependent antibiotic (CDA) and actinorhodin (Act), to disrupt their production in *S. coelicolor* M145 in order to increase prodiginine (Red) production. After pFDZ100-CDA-tandem conjugation into *S. coelicolor* M145, parts of the CDA biosynthetic gene cluster containing NRPS-encoding genes *cdaPS1*, *cdaPS2* and part of *cdaPS3* (*sco3228∼sco3232*, ∼42 kb), were replaced by the apramycin resistance gene, *aac(3)IV*. Double-crossover colonies were obtained by screening the Apra^r^ Thio^s^ exconjungants to generate the mutant strain ZB1 (see Table 1). We then transferred the marker-removal plasmid pZB101, which expresses the φC31 integrase, into ZB1 by conjugation. As shown in Fig. 2B, the resistance gene was flanked by *attB_0_*-φC31 and *attP_0_*-φC31. After *in vivo* site-specific recombination between these two sites catalyzed by φC31 integrase, the resistance gene in the plasmid pTA0613 would be removed, leaving one *attL_0_*-φC31 in the genome. Because the central dinucleotide *attP_0_*-φC31 sequence was not identical to the wild type *attB* site of φC31 integrase, no reaction occurred between *attP_0_* and *attB* in the genome. Exconjungants were screened in the Thio^r^ Kan^r^ MS media, and after the resistance gene was removed, the mutant strain was named ZB2 (see Table 1). We then constructed the CDA and Act biosynthetic double deficient strain ZB3 by further deleting part of the Act biosynthetic gene cluster (including key genes required for ACT biosynthesis, ranging from *sco5087* to *sco5092*, ∼3.6 kb). After deletion of the resistance gene *aphII* by marker-removal plasmid pZB102, the strain was named ZB4 (see Table 1).

In our previous work, we identified the *rrdA* gene as a TetR family protein gene, which regulated secondary metabolite production in *S. coelicolor* by negative regulation of Red biosynthesis and controlling RedD mRNA abundance [25]. Thus, we used the pFDZ100-*rrdA*-tandem to further knock-out the *rrdA* gene from the mutant strain ZB4 for Red overproduction. The mutated strains before and after removing the resistance gene *aphII* were named ZB7 and ZB8 (see Table 1).

Due to the high efficiency and accuracy of the site-specific recombination reaction, the ratio of positive exconjungants with marker removal was about 50%, and the entire process was very time-efficient. Including the construction of plasmids, the deletion of the gene cluster and removal of selectable markers, the whole process took less than a month. Furthermore, compared to other marker excision systems (Flp, Cre, Dre recombinases) [30], [31], φC31 integrase-mediated recombination is highly directional in the absence of the excisionase. As no resistance marker was preserved in the chromosome, this approach avoided the limitations of selectable resistance markers and was quite convenient for the following gene replacement, especially for multiple gene knock-outs.

### PCR verification of the mutants and phenotypes

**Figure 3:**
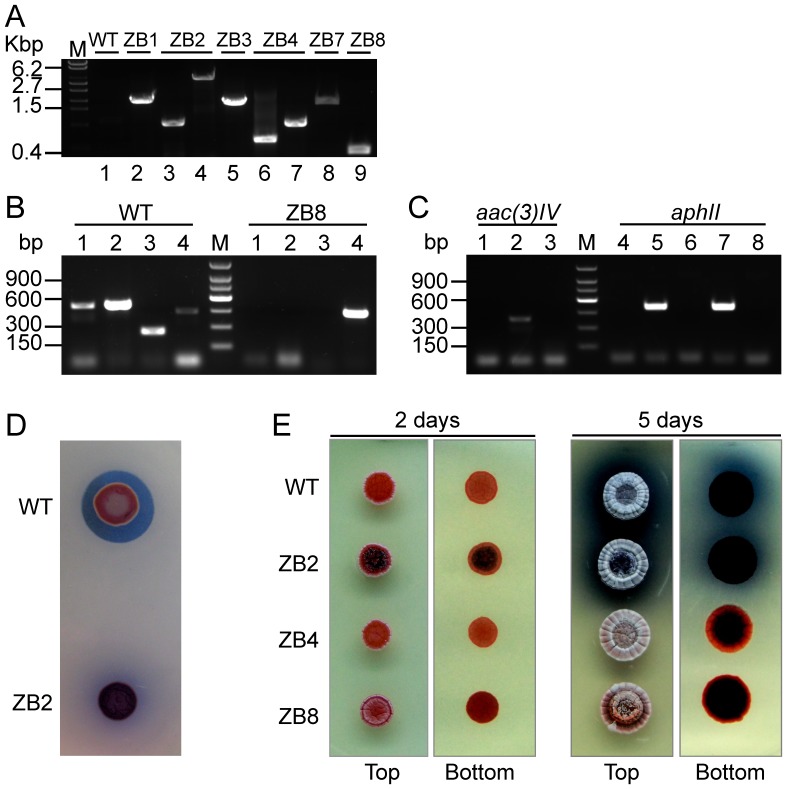
PCR analysis and phenotypes of mutants. (A) PCR verification of gene deletion. Lane 1, a 42,725 bp fragment was amplified using primers ZB287/ZB288. The size of the fragment was too large and could not be PCR-amplified, thus no band was seen in lane 1. Lane 2, a 1981 bp fragment was amplified using primers ZB287/ZB288. The CDA biosynthetic gene cluster was replaced with the *aac(3)IV* gene. Lane3, a 927 bp fragment was amplified using primers ZB287/ZB288, and the resistance gene was removed. Lane 4, a 4073 bp fragment was amplified with primers ZB289/ZB290. Lanes 5 and 6 showed the bands before and after removing the *aphII* resistance gene. Lane 7, a 928 bp fragment was amplified with primers ZB285/ZB286. Lanes 8 and 9 showed thebands before and after removing the *aphII* resistance gene. WT, wild type strain; ZB1∼ZB8, serial mutant strains. (B) PCR verification of gene deletion. Lane 1: the fragment was amplified with primers ZB145/ZB146 and primers were both within *sco3229*. Lane 2: the fragment was amplified with primers ZB195/ZB196 and primerZB195 was within *sco5089* and primer ZB196 was within *sco5090*. Lane 3: the fragment was amplified with primers ZB184/ZB186 and primerZB184 was within *sco1103* and primer ZB184 was within *sco1104*. Lane 4: the fragment as a positive control was amplified with primers ZB147/ZB148 and primers were both within *sco5888*. M: 150 bp ladder. (C) PCR verification of the resistance removing. Lane 1: M145; Lane 2: ZB1; Lane 3: ZB2. The three fragments were amplified with primers ZB189/ZB190, primers were both within the *acc(3)IV* gene. Lane 4: M 145; Lane 5: ZB3; Lane 6: ZB4; Lane 7: ZB7; Lane 8: ZB8. The five fragments were amplified with primers ZB187/ZB188, primers were both within the *aphII* gene. M: 150 bp ladder. (D) Bioassay of CDA extracts from the WT strain and CDA-null mutant strain ZB2. (E) Phenotypes of wild-type and three mutant strains. The spores were cultured for 2 days (left) and 5 days (right) on R2YE agar.

Each null mutant was verified by PCR amplification using a pair of oligonucleotide primers specific for the flanking chromosomal DNA sequences of the target gene. The results are shown in Fig. 3A. In lane 1, no band was seen because the PCR fragment size was very large (∼42 kb), which almost contained the entire CDA biosynthesis gene cluster, and could not be amplified. The further verification was also carried on by PCR amplification using two different pairs of oligonucleotide primers. One pair of primers was synthesized according to the internal sequences of the deleted gene clusters or gene, and the other one was based on the internal sequences of the resistance gene. The results were shown in Fig. 3B and Fig. 3C. No band was amplified with the former primers and a 500 bp-size band could be seen clearly with the later primers in the double-crossover null mutant. It turned out just the opposite result in the wild type *Streptomyces coelicolor* M145. The confirmation of removing the resistance gene was seen in Fig. 3A and Fig. 3C. The PCR products had also been sequenced to ensure excision of the antibiotic cassettes occurred precisely (Fig. S1). The *aac(3)IV* gene was used as a selectable marker in ZB1 while using *aphII* in ZB3 and ZB7, due to cross-resistance (<50 µg/ml) of the *aac(3)IV* gene to kanamycin that could have complicated the marker-removal process by false positives. However, almost no cross-resistance (<5 µg/ml) of the *aphII* gene to apramycin occurred.

Mutant phenotypes are shown in Fig. 3D and 3E. CDA production analysis was carried out in line with previous descriptions [25], [27]. *B. mycoides* was used as an indicative strain to detect CDA activity. First, spores were spotted onto oxoid nutrient agar plates, air-dried and cultured for 36∼48 h in a 30°C incubator. Then, the plate was overlaid with the soft nutrient agar containing *B. mycoides* and Ca(NO_3_)_2_ at a 12 mM final concentration and CDA activity was detected after one day. CDA produced by wide-type M145 could kill *B. mycoides* cells, thus a clear inhibition zone was seen on the plate, but no inhibition zone was observed during CDA biosynthesis by the mutant (see Fig. 3D).

The phenotypes of the wild-type strain and the mutants ZB2, ZB4 and ZB8 are exhibited in Fig. 3E. The loss of blue pigment production indicated that Act biosynthesis was disrupted. The deletion of the *rrdA* gene was shown by an increase of red pigment production (at 2 and 5 days).

### Analysis of growth and prodiginine (Red) production

To assess mutant effects on the growth and development of the different strains, each mutant and the parent strain were spotted onto the rich R2YE agar medium plate, then spores were incubated at 30°C for 2 to 5 days to test the growth performance. All strains exhibited no apparent differences in aerial mycelia formation or sporulation (Fig. 3E). Strain ZB4 and ZB8 failed to produce Act.

**Figure 4:**
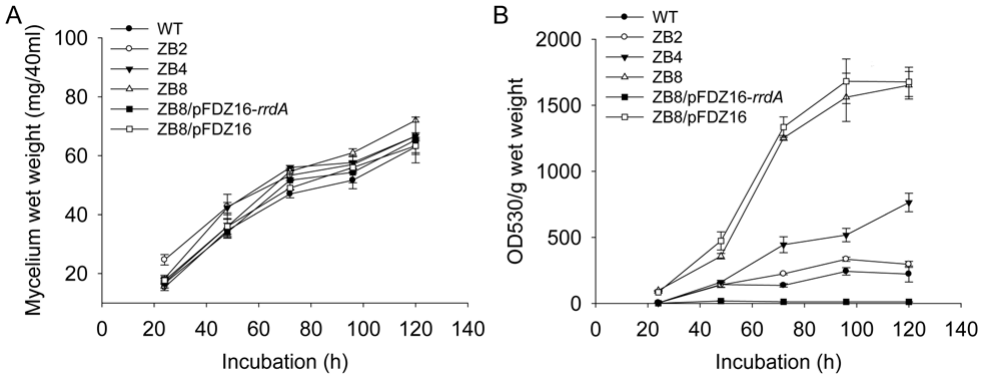
Growth curves and prodiginine production of wild-type strain, three mutant strains and complemented strains. Growth curves (A) and prodiginine production (B) of M145, ZB2, ZB4, ZB8, ZB8/pFDZ16 and ZB8/pFDZ16-*rrdA* growth in 40 ml of R4 liquid medium. Incubation was carried out at 30°C. The symbols indicate the averages of three independent determinations and the error bars indicate the standard errors. OD_530_, optical density at 530 nm.

The wild-type and mutated strains were cultured in 40 ml of R4 liquid medium to investigate the growth rate and prodiginine production quantitatively. Spores were inoculated in a reciprocal shaker (180 rpm) at 30°C with an inoculation of 2×10^7^spores per ml. The mycelia were harvested at different time points and treated as described in the Materials and Methods section. As shown in Fig. 4A, no statistical significance in growth rate was observed among all the strains, and the mutated strains (ZB2, ZB4 and ZB8) produced more prodiginine than the wild-type strain (Fig. 4B). Red overproduction might be attributable to competition for common precursors of CDA, Act and Red biosynthesis [25]. Red production of the double null mutant strain ZB4(ΔCDA ΔAct) was approximately 3.5 times that of the wild-type strain, and this further suggested competition for common precursors of the three biosynthetic pathways. Furthermore, our previous study [25] showed that Red production of the *rrdA-*null mutant was two-fold higher than that of the wild-type strain by releasing the negative control of *redD* mRNA abundance. In the present study, Red production in strain ZB8 was over five-fold higher than the wild-type strain (Fig. 4B). In order to illustrate the important role of *rrdA* more clearly, we tried to overexpress *rrdA* in ZB8 strain by introducing the plasmid pFDZ16-*rrdA* and red production showed much lower level than wild-type strain. However, the complementation strain harboring the empty vector pFDZ16 produced almost the same prodiginine production as ZB8 strain (Fig. 4B). Thus, the *rrdA* as a negative regulator of Red production played an important role. The Red-overproducing strain ZB8 could be rapidly constructed using our method.

In conclusion, we described a rapid and efficient method for marker-free *S. coelicolor* mutagenesis by site-specific recombination. This approach was time-saving and facilitated multiple deletion rounds in the *Streptomyces* genome. The utility of this method was clearly demonstrated by construction of a prodiginine (Red) overproducing strain, ZB8, which produced five times the amount of Red compared to the wild-type strain. This strategy provided an alternative approach for rapid markerless modification of the actinobacterial genome.

## Supporting Information

Table S1 **Primers used in this study.**
(DOCX)

Text S1 **Construction of plasmids pXD34-int, pFDZ100-CDA-tandem, pFDZ100-Act-tandem and pFDZ100-** ***rrdA*** **-tandem.**
(DOCX)

Figure S1 **Sequence analysis of PCR products for verification of the markerless mutants.** Primers ZB469 (for ZB2), ZB472 (for ZB4) and ZB473 (for ZB8) were used for sequencing the PCR products.
(TIFF)
