# Supplementary material for: An Efficient Procedure for Marker-Free Mutagenesis of S. coelicolor by Site-Specific Recombination for Secondary Metabolite Overproduction
Source: PLoS One. 2013 Feb 7;8(2):e55906. doi: 10.1371/journal.pone.0055906 (PMC3567011; doi:10.1371/journal.pone.0055906)
Supplement: Table S1 — Primers used in this study. (DOCX) [file pone.0055906.s001.docx]

**Table S1. Primers used in this study.**

| Primers | Sequences |
| --- | --- |
| ZB125 | 5'-GCCACCATCTCCGCCACCT-3' |
| ZB126 | 5'-ACCGCAGCTTCCGCTCCCT-3' |
| ZB127 | 5'- TGGTCTGCCTGGCTCGTA-3' |
| ZB128 | 5'- CGGACGGCTCGGGATGAT-3' |
| ZB129 | 5'- CGCTCTGACCACCAGGGAA-3' |
| ZB130 | 5'- AGCCACGGATGACCACCAC-3' |
| ZB131 | 5'-CCGACACCCAGGTCAAGC-3' |
| ZB132 | 5'- GCGACTCCAGGTAGCCGTA-3' |
| ZB145 | 5' -CAGGGCCTTGATGTTGGA-3' |
| ZB146 | 5' -GAGCACCGCCGAGTTCTA-3' |
| ZB147 | 5'-CGGCCTCGGTTCCTGTCTG-3' |
| ZB148 | 5' -AGCGGTCCTGCGGGTTGA-3' |
| ZB153 | 5' -ACACTGGTTCATGTGCAGC-3' |
| ZB154 | 5'-AGTACGCGCCCGGGGAGCCCACGGGCACGCCCTGGCACCCGCCCAGCACTCGAATGGCTCA-3' |
| ZB155 | 5'-GTAGTGCCCCAACTGGGGTAACCTGTGAGTTCTCTCAGTTGGGGGCGTAGCCAGCTCTACACTGGTTCA-3' |
| ZB156 | 5' -CGAATGGCTCAGCCAATCG-3' |
| ZB180 | 5' -GAGGATCGCAGCATGCAGAC-3' |
| ZB181 | 5' -TGCTGTACTCCTGCGTTGAC-3' |
| ZB182 | 5' -CCGAGTTGTTCGAGCGCTAC-3' |
| ZB183 | 5' -CATGGCGAAGACGATACTGC-3' |
| ZB184 | 5' -TGCACTTCGTGGACCACTTG-3' |
| ZB186 | 5' -CGCCGCAATTCTTCATTGAC-3' |
| ZB187 | 5' -CGACGTTGTCACTGAAGCGGGAAGG-3' |
| ZB188 | 5' -CGGCGATACCGTAAAGCACGAGGAA-3' |
| ZB189 | 5' - GGTCCACAGCTCCTTCCGTAGCGT-3' |
| ZB190 | 5'- GTCGTGTTGGCATCGTGTCCCA-3' |
| ZB195 | 5'-AGCTGCTCGACCTGATCAAC-3' |
| ZB196 | 5' -AACGTGAAGACCAGCTCCTC-3' |
| PTB15 | 5'-GATCTAGAGCTGGATCATCTGGATCGGTTTCGTCAAAAACCTGGCCGAATAAATACCTGTGAC-3' |
| PTP00 | 5'-GCGGATCCCGGTGCTGGGTTGTTGTCTCTGGACAGTGATCCATGGGAAACTACTCAGCACCTACGCCCCGCCCTGCCACT-3' |
| PTB13 | 5'-GACCAGGTTTTTGACGAAACAGATCCAGATGATCCAGCTCTACACTGGTTCATGTGCAGCTCCATC-3' |
| PTP15 | 5'-GGTGCTGAGTAGTTTCCCATGGATCGGTGTCCAGAGACAACAACCCAGCACTCGAATGGCTCAGCCAATCGACTGGC-3' |
| PTB00 | 5'-GACCAGGTTTTTGACGAAAGTGATCCAGATGATCCAGCTCTACACTGGTTCATGTGCAGCTCCATC-3' |
| PTP06 | 5'-GGTGCTGAGTAGTTTCCCATGGATCAGTGTCCAGAGACAACAACCCAGCACTCGAATGGCTCAGCCAATCGACTGGC-3' |
| PTB06 | 5'-GACCAGGTTTTTGACGAAACTGATCCAGATGATCCAGCTCTACACTGGTTCATGTGCAGCTCCATC-3' |
| PTP13 | 5'-GGTGCTGAGTAGTTTCCCATGGATCTGTGTCCAGAGACAACAACCCAGCACTCGAATGGCTCAGCCAATCGACTGGC-3' |
| Oxj128 | 5' -GGAAGGGCAGCCCACCTA-3' |
| Oxj129 | 5' -CCCCAGAGTCCCGCTCAG-3' |
| ZB285 | 5'-GCACTTCGTGGACCACTTGC-3' |
| ZB286 | 5'-GCAGCCGGACTACTTCGACT-3' |
| ZB287 | 5'-CAGGTGGTCGAGGTAGGTCT-3' |
| ZB288 | 5'-CCGGAGTAGCACCAGCTCAT-3' |
| ZB289 | 5'-GCCGAGCAGGATGATGTGGT-3' |
| ZB290 | 5'-CACGAGACAGCGGCGTACAA-3' |
| ZB291 | 5'-GGAGCGGACATATGAAATACCTGCTGCCG-3' |
| ZB292 | 5'-TAGCTAGCCTACGCCGCTACGTCTTC-3' |
| ZB469 | 5'-CAGGTGGTCGAGGTAGGTCT-3' |
| ZB472 | 5'-GCCGAGCAGGATGATGTGGT-3' |
| ZB473 | 5'- TGCACTTCGTGGACCACTTG-3' |

Underlined text in ZB154 and ZB155 primer sequences represents the φC31 integrase recognition sites *attB_0_*-φC31 and *attP_0_*-φC31, and other underlined text in primer sequences represents the *attB* and *attP* recombination sequence of the φBT1 integrase.
