# Supplementary material for: An Efficient Procedure for Marker-Free Mutagenesis of S. coelicolor by Site-Specific Recombination for Secondary Metabolite Overproduction
Source: PLoS One. 2013 Feb 7;8(2):e55906. doi: 10.1371/journal.pone.0055906 (PMC3567011; doi:10.1371/journal.pone.0055906)
Supplement: Text S1 — Construction of plasmids pXD34-int, pFDZ100-CDA-tandem, pFDZ100-Act-tandem and pFDZ100- rrdA -tandem. (DOCX) [file pone.0055906.s002.docx]

***Construction of the plasmids***

**Construction of plamid pXD34-int**

Plasmids pIJ6021 and pSET152 were digested with BamHI and SphI respectively, blunted by Klenow Fragment (TaKaRa, Kyoto, Japan), and further digested with EcoRI to generate an 8.2-kb fragment and a 3.1-kb fragment; the two fragments were ligated to obtain the plasmid pXD34, pXD34 was further digested with NdeI/XbaI. A PCR fragment containing φC31 integrase gene was amplified from pSET152 using primers ZB291/ZB292, digested with NdeI/NheI, and then inserted into the linearized pXD34, to yield plasmid pXD34-int.

**Construction of pFDZ100-CDA-tandem, pFDZ100-Act-tandem and pFDZ100-*rrdA*-tandem**

To construct pFDZ100-CDA-tandem, plasmids pFDZ101-CDA-5’arm, pTA0613, and pFDZ103-CDA-3’arm were digested with EcoRI and HindIII to generate three linear fragments which flanked by different *attB* and *attP* recombination sites; then the three fragments and plasmid pFDZ100 were incubated with φBT1 integrase in proper buffer condition (see Materials and Methods). The reaction products were transformed into *E.coli* and selected by proper antibiotics, the positive clones were propagated and the plasmids were isolated to be pFDZ100-CDA-tandem.

Same strategies were employed to construct pFDZ100-Act-tandem and pFDZ100-*rrdA*-tandem, except the four entry plasmids were different. Plasmids pFDZ101-Act-5’arm, pFDZ102, pFDZ103-Act-3’arm and pFDZ100 were used for construction of pFDZ100-Act-tandem; and pFDZ101-*rrdA*-5’arm, pFDZ102, pFDZ103-*rrdA*-3’arm and pFDZ100 were used for construction of pFDZ100-*rrdA*-tandem.
