# Supplementary figures and images for: An Efficient Procedure for Marker-Free Mutagenesis of S. coelicolor by Site-Specific Recombination for Secondary Metabolite Overproduction
Source: PLoS One. 2013 Feb 7;8(2):e55906. doi: 10.1371/journal.pone.0055906 (PMC3567011; doi:10.1371/journal.pone.0055906)

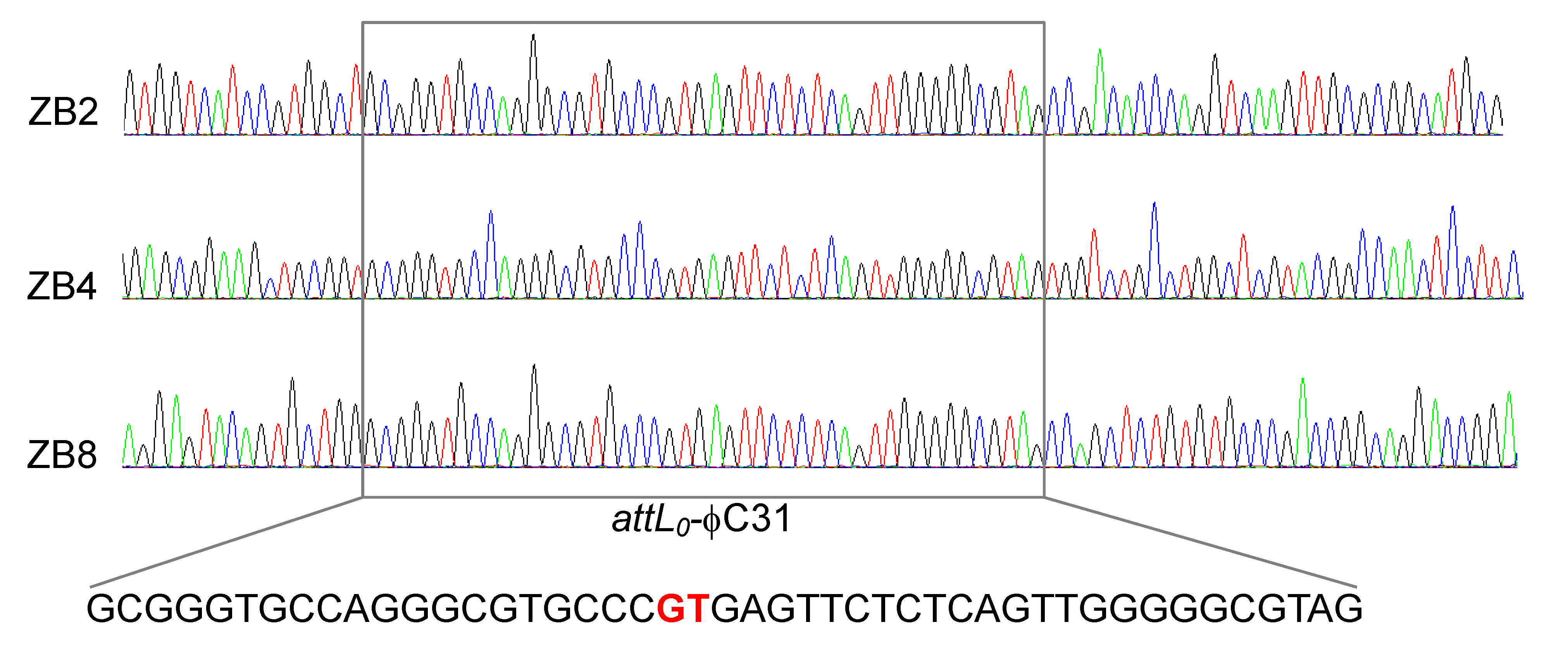

Supplement: Figure S1 — Sequence analysis of PCR products for verification of the markerless mutants. Primers ZB469 (for ZB2), ZB472 (for ZB4) and ZB473 (for ZB8) were used for sequencing the PCR products. (TIFF) [file pone.0055906.s003.tif]
